# PNIPAM Mesoglobules in Dependence on Pressure

**DOI:** 10.1021/acs.langmuir.4c02952

**Published:** 2024-10-12

**Authors:** Bart-Jan Niebuur, Vitaliy Pipich, Marie-Sousai Appavou, Dharani Mullapudi, Alec Nieth, Eric Rende, Alfons Schulte, Christine M. Papadakis

**Affiliations:** † TUM School of Natural Sciences, Physics Department, Soft Matter Physics Group, Technical University of Munich, James-Franck-Str. 1, Garching 85748, Germany; ‡ Jülich Centre for Neutron Science (JCNS) at Heinz Maier-Leibnitz Zentrum (MLZ), Forschungszentrum Jülich GmbH, Lichtenbergstr. 1, Garching 85748, Germany; § Department of Physics and College of Optics and Photonics, University of Central Florida, 4111 Libra Drive, Orlando, Florida 32816-2385, United States

## Abstract

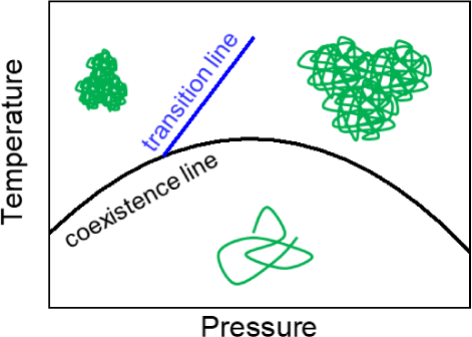

Poly(*N*-isopropylacrylamide) (PNIPAM) in aqueous solution forms mesoglobules above its cloud point temperature *T*_cp_. While these are small and compact at atmospheric pressure, they are large and water-rich at high pressure. To identify the transition between these states, we employed optical microscopy and carried out isothermal pressure scans. Using very small angle neutron scattering, we determined the size and water content of the mesoglobules in pressure scans at different temperatures above *T*_cp_. We observe a distinct transition at pressures of 35–55 MPa with the transition pressure depending on temperature. While the transition is smooth at high temperatures, i.e., far away from the coexistence line, it is abrupt at low temperatures, i.e., close to the coexistence line. Hence, at high temperatures, the swelling of the mesoglobules dominates, whereas at low temperatures, the coalescence of mesoglobules prevails. Subsequently decreasing the pressure results in a gradual deswelling of the mesoglobules at high temperature. In contrast, at low temperatures, small and compact mesoglobules form, but the large aggregates persist. We conclude that, on the time scale of the experiment, the disintegration of the large swollen aggregates into small and compact mesoglobules is only partially possible. Erasing the history by cooling the sample at the maximum pressure into the one-phase state does not result in qualitative changes for the behavior with the only difference that Fewer mesoglobules are formed when the pressure is decreased again. The newly identified transition line separates the low-pressure from the high-pressure regime.

## Introduction

Stimuli-responsive polymers are important from a fundamental perspective as well as for applications, such as drug delivery systems, templates for tissue engineering, sensors and switches.^1−3^ These extend to stimuli-responsive polymer interfaces^4^ and thin films (ref. (5) and references therein). A prominent class are thermoresponsive polymers with a lower critical solution temperature (LCST) in aqueous solution. These are soluble below the cloud point temperature *T*_cp_ and insoluble above.^6^ The model system poly(*N*-isopropylacrylamide) (PNIPAM) features LCST behavior in aqueous solution while undergoing a coil-to-globule transition with a *T*_cp_ near 32 °C at ambient pressure over a wide range of concentrations.^7,8^ The collapsed and water-insoluble polymers form aggregates, which are long-lived and of mesoscopic size, with radii typically in the submicrometer range.^9−16^ They are commonly named “mesoglobules”. They expose a dense and rigid PNIPAM shell, and their water content is rather low, as found in our previous studies.^17^ Time-resolved experiments have revealed that their coalescence is impeded by the viscoelastic effect, associated with the polymer-rich, rigid PNIPAM shell covering the mesoglobules. It may be at the origin of their longevity.^18,19^ Hence, macrophase separation is severely delayed.

Pressure has a significant effect on the water solubility and the degree of hydration of PNIPAM among other polyacrylamides. The coexistence line of aqueous solutions of PNIPAM in the temperature–pressure frame has the shape of an ellipse,^18,20−24^ i.e., *T*_cp_ increases with pressure by a few degrees, before it decreases again. Inside the ellipse, i.e., at low temperatures and pressures, resides the one-phase state, while the solution is in the two-phase state outside. The change in slope of the coexistence line has been attributed to the change in molar volume by mixing, which initially decreases with increasing pressure. At higher pressure, hydrogen bonding may be promoted^25^ and ordered, clathrate-like water cages form around the hydrophobic groups, resulting in a positive change of mixing volume and hence a decrease of *T*_cp_.^26,27^ A recent review of the pressure-dependent behavior of aqueous PNIPAM solutions is given in ref. (28)

For a 3 wt % PNIPAM solution in D_2_O, the cloud point at atmospheric pressure is 33.6 °C, while the maximum of the ellipse is located at ca. 60 MPa and 36 °C.^18^ The size and water content of the mesoglobules depend strongly on pressure: Whereas heating the PNIPAM solution to the two-phase state at atmospheric pressure results in small and compact mesoglobules having a radius of about 0.5 μm, large, water-rich aggregates with radii of 1–2 μm are formed at 80 and 113 MPa.^17^ The same structures were observed after isothermal pressure jumps from the one-phase to the two-phase state.^18,19^ We refer to these distinct states as the low- (LP) and the high-pressure (HP) regime. In recent atomistic simulations of a single PNIPAM chain in aqueous solution, collapsed chains (“globules”) with a low degree of hydration were found above the coexistence line at pressures up to ca. 200 MPa.^29^ In contrast, at pressures above this value, the chains are collapsed as well, but they retain a high degree of hydration, and the hydration shell is more structured than at low pressures.^29^

While the mesoglobules have been studied experimentally in temperature scans at low and at high pressure,^17^ the nature of the transition from small and compact mesoglobules to larger, water-rich aggregates and back has not been characterized. In this regard, we are only aware of a study on the volume changes of chemically cross-linked PNIPAM hydrogels^30^ and of a pressure jump study on poly(*N*-*n*-propylacrylamide) microgels.^31^

At a temperature above the maximum of the coexistence line, the volume of the hydrogels^30^ increases gradually by a factor of ∼2, as pressure is increased from 100 to 200 MPa. We anticipate that, in our solution of linear PNIPAM homopolymers, the transition from small mesoglobules to large aggregates may proceed via swelling of the mesoglobules by the uptake of water or by their merging via coagulation or coalescence. Regarding the backward transition, compact regions are expected to form by chain collapse and the disintegration of these collapsed regions. For both processes, the water solubility and the mobility of the PNIPAM chains—which depends on the degree of hydration of the PNIPAM chains—appear to play an important role. Both factors are a function of the temperature- and pressure-distances to the coexistence line in the two-phase region.

Here, we present structural studies of the size and water-content of the mesoglobules formed in a semidilute aqueous solution of PNIPAM in dependence on pressure in the two-phase state, i.e., at temperatures above the coexistence line. We chose the same polymer concentration (3 wt %) as in our previous investigation, where we carried out temperature scans at pressures between 0.1 and 113 MPa,^17^ and performed isothermal pressure scans between 10 and 110 MPa, i.e., from the LP to the HP regime and back. Optical microscopy (OM) displays the pressure-dependent size of the mesoglobules. As in our previous study,^17^ microcapillaries are used as sample cells, and are placed in a temperature-controlled holder. Furthermore, very small-angle neutron scattering (VSANS) is leveraged to resolve structures up to a few μm, which enables a quantitative characterization of the sizes of the mesoglobules and their water content. The manuscript is organized as follows: Following the Materials and Methods Section, the results from OM during pressure scans are given. Then, the results from isothermal VSANS pressure scans are presented. Finally, the key parameters for the transition behavior are identified and discussed.

## Materials and Methods

### Materials

Poly(*N*-isopropylacrylamide) (PNIPAM) with a molar mass *M*_n_ = 36 000 g mol^–1^ and a dispersity of 1.26 was purchased from Sigma-Aldrich. It was dissolved at a concentration of 3 wt % in an 80:20 v/v mixture of D_2_O and H_2_O. This polymer concentration is above the overlap concentration, the solution is thus semidilute.^18^ The D_2_O:H_2_O mixing ratio was chosen to reduce multiple scattering in VSANS measurements, while ensuring sufficient contrast between water-rich and polymer-rich phases.^32^ The scattering length densities (SLDs) of PNIPAM and the D_2_O/H_2_O mixture are 0.814 × 10^–6^ Å^–2^ and 4.97 × 10^–6^ Å^–2^, respectively. Compared to a 3 wt % PNIPAM solution in neat D_2_O, as studied by us before,^18^ the coexistence line is expected to be shifted to lower temperatures by ca. 0.5 °C.^23^

### Methods

Optical imaging at variable pressure and temperature was conducted with a home-built system based on an Olympus X41 microscope and a CMOS camera, which was described previously.^17^ The sample solutions were contained in a fused silica microcapillary high pressure cell (lateral size 100 μm), withstanding pressures up to 300 MPa.^17,33^ It was connected to a pressure generator (High Pressure Equipment Company), using ethanol as the pressure transmitting medium. The sample solution was probed near the end of the capillary (∼30 cm away from the sample-ethanol interface) so that no contamination of the sample with ethanol could occur.^34^ The capillary was anchored in a Cu block that was attached to a circulating bath thermostat, for good thermal contact and temperature homogeneity. The temperature was measured with a Pt100 resistance thermometer in the Cu block. A possible small temperature offset was corrected by comparing with *in situ* microturbidimetry.^17^ After each temperature change, the sample was equilibrated for 10 min, and after each pressure change for 2 min. Image sequences were recorded with the CMOS camera employing a 50× Olympus X41 microscope.

**Figure 1:**
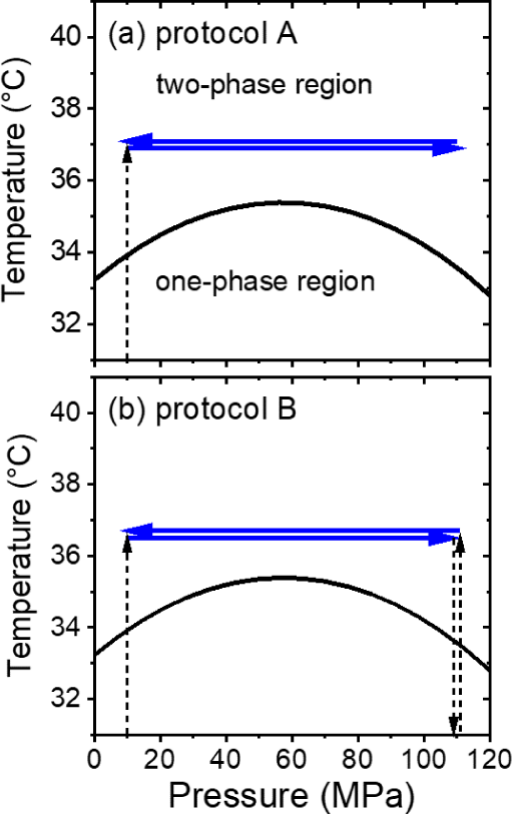
Schematic representation of protocols A (a) and B (b) for OM and VSANS. The coexistence line is taken from our previous measurements of a 3 wt % PNIPAM solution in D_2_O, which was shifted downward by 0.5 °C to account for the change of solvent to H_2_O:D_2_O.^18^ Blue full arrows denote the scans carried out. Black dashed lines indicate changes of temperature or pressure without measurements being taken on the way.

For the characterization of the overall sizes of the mesoglobules/aggregates by OM experiments, the protocols shown in Figure 1a (protocol A) and b (protocol B) were followed. In detail, for protocol A, the sample was heated to 37.0 °C at a pressure of 14 MPa, and after an equilibration time of 10 min, the pressure was increased to 110 MPa in steps of 7 MPa. For protocol B, a pressure scan was carried out by heating the sample to 36.6 °C at a pressure of 35 MPa, and after an equilibration time of 10 min, the pressure was increased stepwise to 110 MPa. Before decreasing the pressure again, the sample was cooled to 25 °C, i.e., deep into the one-phase state and was equilibrated for 10 min. The sample was reheated to 36.6 °C, and after an equilibration time of 10 min, the pressure was decreased again in steps.

*Very Small Angle Neutron Scattering (VSANS)* measurements were performed at the instrument KWS-3 at the Heinz Maier-Leibnitz Zentrum (MLZ), Garching, Germany.^35^ Using a neutron wavelength λ = 12.8 Å with a spread Δλ/λ = 0.18 and a sample–detector distance of 9.4 m, a *q* range of 1.4 × 10^–4^ – 2.2 × 10^–3^ Å^–1^ was covered. The sample was mounted in a temperature-controlled custom-made pressure cell based on the one described in ref. (36) which is capable of withstanding pressures up to 500 MPa. The sample was placed between sapphire windows and had a thickness of 1 mm, independent of pressure. The temperature of the pressure cell was controlled using a Julabo heating bath and was measured by a sensor in the CuBe-body of the pressure cell.

The structures of the mesoglobules were characterized in isothermal pressure scans, as depicted schematically in Figure 1. The sample was heated at a pressure of 10 MPa from 20 °C to 35.4, 36.2, or 37.0 °C. After an equilibration time of 2 h, measurements were performed during pressure scans from 10 to 110 MPa and back in steps of 10 MPa (protocol A, Figure 1a). Furthermore, the sample was heated at a pressure of 10 MPa from ∼20 to 35.4 °C. After an equilibration time of 2 h, measurements were performed during pressure scans from 10 to 110 MPa in steps of 10 MPa. Afterward, the sample was cooled to 28.8 °C for 1 h, i.e., deep into the one-phase state. After an equilibration time of 1 h, the sample was heated again to 35.4 °C, and, after a waiting time of 1 h, the pressure was decreased in steps of 10 MPa back to 10 MPa (protocol B, Figure 1b).

In all scans, the sample was equilibrated for 5 min after each change of pressure, followed by 5 measurements of 5 min each. In all cases, the obtained scattering curves overlapped and were averaged. The background was determined from a measurement of the sample solution at 10 MPa and 20 °C. The dark current was measured using boron carbide. Both contributions were subtracted from the data. A Plexiglas standard measurement was used to determine the detector sensitivity, and the intensity of the beam measured with the empty cell was used to bring the data to absolute scale. It was measured directly on the detector. These operations as well as azimuthal averaging of the 2D detector data were carried out using the software QtiKWS by JCNS. For all pressure scans and pressures above 10 MPa, the neutron transmission values are higher than 0.5. Thus, multiple scattering is negligible.

The VSANS scattering profiles were analyzed by least-squares fits to structural models. Scattering curves of the sample exhibiting a shoulder were modeled using the empirical Beaucage model^37,38^ because of its simplicity, along with an incoherent background:
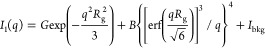
1

Here, *G* and *B* are scaling factors and *R*_g_ the radius of gyration of the mesoglobules/aggregates, respectively. erf(*x*) denotes the error function. In all cases, the background *I*_bkg_ was kept fixed during fitting at 300 cm^–1^.

In some cases, two shoulders were observed in the scattering data, which point to the coexistence of large and small structures. These data were modeled by the sum of two Beaucage functions:
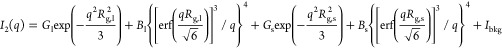
2where *G*_l_, *B*_l_, *G*_s_ and *B*_S_ are scaling factors (*l* and *s* stand for large and small, respectively), and *R*_g,l_ and *R*_g,s_ are the radii of gyration of the large and small structures.

In all cases, the scattering invariant Q* was calculated as
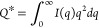
3using the *q*-dependent terms of the respective fitting function, but excluding *I*_bkg_. For a two-phase system, Q* reads
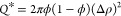
4where ϕ is the volume fraction of the polymer-rich domains and Δρ the difference in SLDs between the two phases.

Some of the scattering curves exhibit a decay that does not have a shoulder but rather is straight in the log–log-representation. In these cases, the aggregates are so large, that their size cannot be determined with the measured *q*-range. These curves were modeled by the Porod approximation together with an incoherent background:
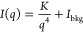
5The Porod constant *K* is related to the specific surface of the aggregates, *S*/*V*, by
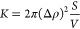
6

Standard procedures were applied to account for the divergence of the neutron beam and the wavelength distribution.^39^ Fitting routines were implemented in Python.

## Results and Discussion

In this section, we first present results from OM during pressure scans and then discuss the VSANS results from the three protocols described in the Methods section.

### Structural Studies Using Optical Microscopy

**Figure 2:**
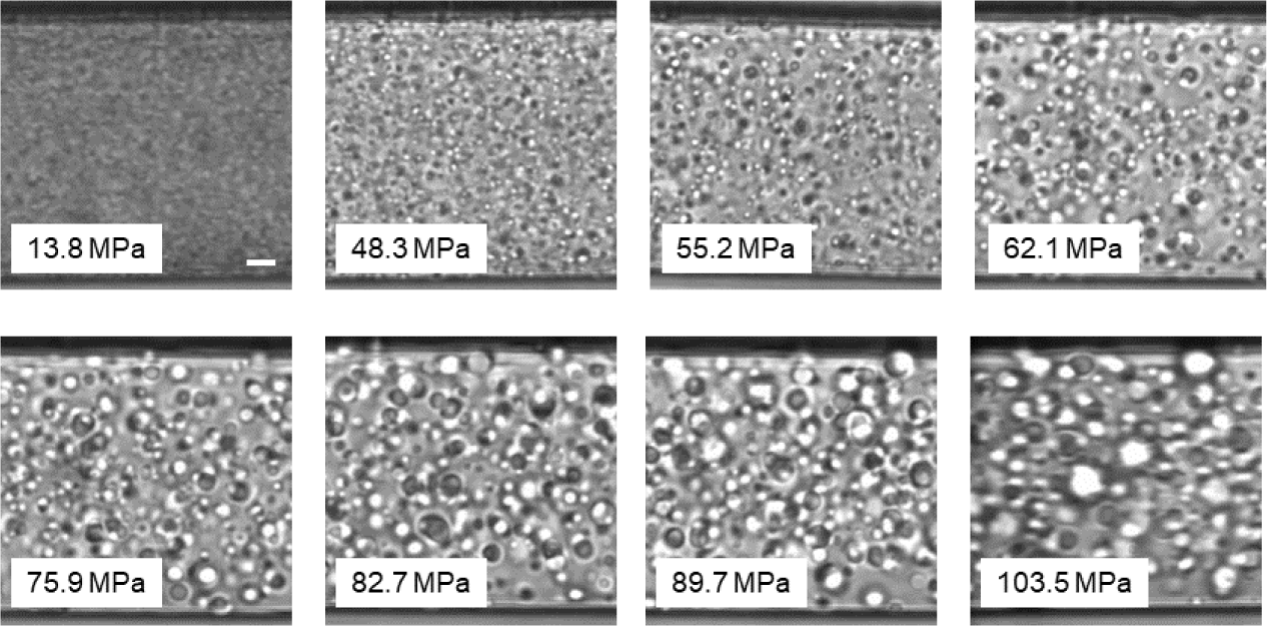
Representative images from OM at 37.0 °C during a pressure increase along the trajectory of protocol A. The scale bar given in the image taken at 13.8 MPa corresponds to a length of 10 μm.

Optical microscopy provides first insights into the temperature- and pressure-dependent structures in a semidilute PNIPAM solution in the two-phase state. Figure 2 shows images taken during a pressure increase at 37.0 °C, which is above the maximum temperature of the coexistence line (protocol A). With pressure increasing from 13.8 to 103.5 MPa, the average radius of the mesoglobules increases from 1.2 to 2 μm. We note that, especially at high pressures, some of the mesoglobules may have adhered to the glass wall of the capillary, which makes them look larger. These were excluded from the analysis.

**Figure 3:**
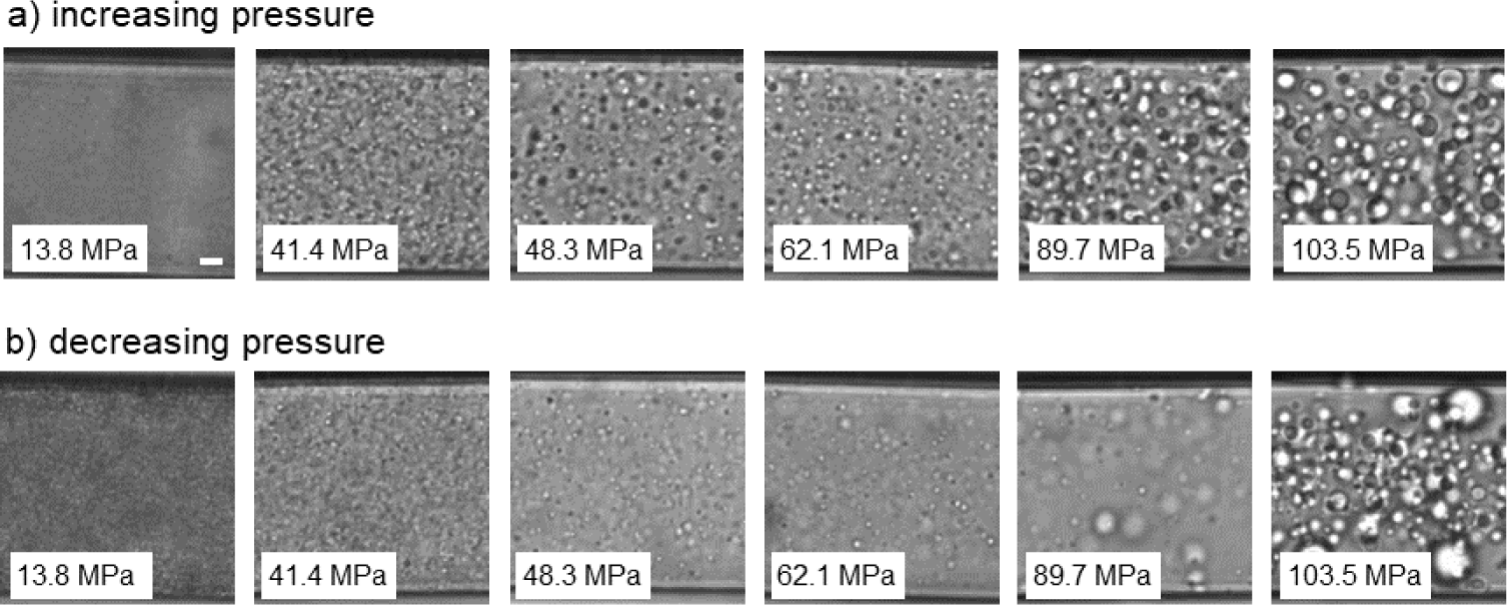
Representative images from OM at 36.6 °C (protocol B) with increasing (left to right) (a) and decreasing pressure (right to left) (b). The scale bars given in (a) correspond to a length of 10 μm.

Another pressure increase scan was carried out at a slightly lower temperature, namely at 36.6 °C, following protocol B (Figure 1b). The optical images are shown in Figure 3a. Again, a transition from smaller mesoglobules to larger aggregates is observed. After reaching the maximum pressure, the sample was cooled to 25 °C, i.e., to the one-phase state, to dissolve possible traces of mesoglobules that may have an influence on the behavior during a pressure decrease. Subsequently, it was heated again to 36.6 °C, and the pressure was decreased stepwise to 13.8 MPa (Figure 3b). The size of the mesoglobules shrinks again, i.e., the transition is reversible.

### Structural Studies Using VSANS

To elucidate the mesoglobule structures at submicron spatial resolution and higher statistical relevance, we conducted VSANS measurements during similar isothermal pressure scans. Protocols A and B were used to characterize the behavior at different temperatures above the coexistence line (Figure 1).

#### Pressure Scans Following Protocol A

**Figure 4:**
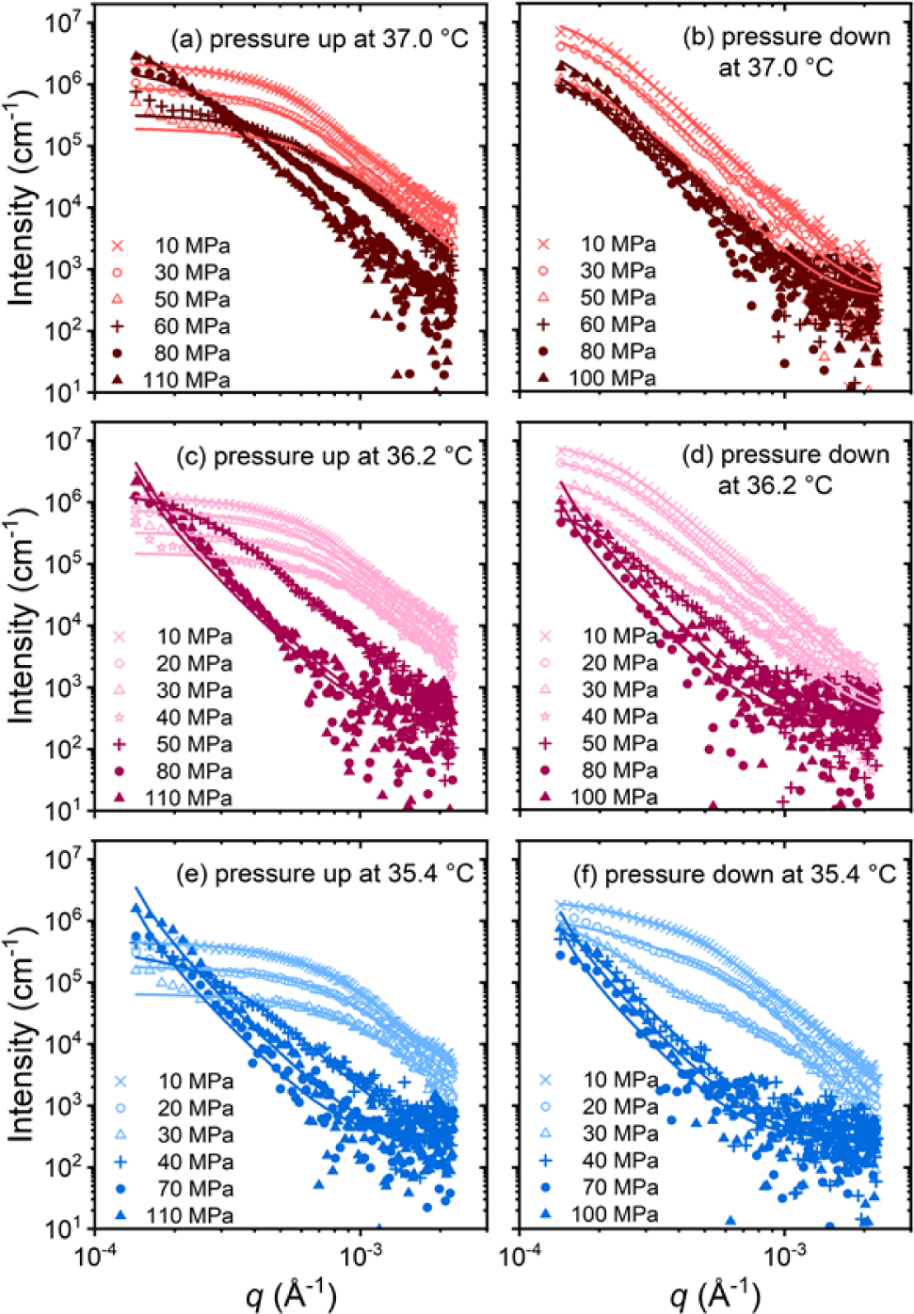
Representative VSANS curves obtained during protocol A during a pressure increase from 10 to 110 MPa and back at 37.0 °C (a, b), 36.2 °C (c, d) and 35.4 °C (e, f). Symbols: experimental data. Solid lines: fits of 1 or 2 Beaucage form factors or of the Porod form factor (eqs 1, 2 and 5, respectively). Open and closed symbols indicate the regimes deduced from the increase in *R*_g_ (Figure 5 below).

To probe the transition between small mesoglobules in the LP regime and large aggregates in the HP regime observed in OM, protocol A was executed. Three temperatures were chosen (37.0, 36.2, and 35.4 °C) to elucidate the effect of the proximity of the coexistence line. Figure 4 displays representative scattering curves. These cover a *q*-range, that corresponds to length scales of ∼0.2–2 μm, which matches the typical size of the small mesoglobules in the LP regime and large aggregates in the HP regime, as found by OM. The scattering from concentration fluctuations inside the mesoglobules and from single dissolved chains is outside the *q*-range covered, i.e., these contributions are not observed.

Figure 4a shows the scattering curves at 37.0 °C during the pressure increase. At 10 MPa, a shoulder is discernible. With increasing pressure, the overall intensity decreases, which points to an increased hydration of the mesoglobules, leading to a loss of scattering contrast. Between 60 and 70 MPa, the shoulder abruptly transforms into a nearly straight line in the double-logarithmic representation, and this shape persists up to 110 MPa. Thus, a transition from small and compact mesoglobules having a size of a few 100 nm to large aggregates having a size in the micrometer range is observed. We attribute this size increase tentatively to a swelling of the mesoglobules and/or their coalescence. When pressure is decreased again, the transition is observed as well, but it is not as pronounced (Figure 4b). The original shape observed before the pressure increase is not fully recovered. At 36.2 °C (Figure 4c,d), similar behavior is observed, however, the overall intensity is lower, the change in curve shape is shifted to lower pressures (40–50 MPa), and it is more pronounced. Moreover, at pressures of 70 MPa and above, the shoulder transforms into a straight decay. Also at this temperature, the transition is not fully reversible, but, below the transition pressure, an additional shoulder appears at high *q*-values (above ca. 6 × 10^-4^ Å^–1^), which grows in intensity. At 35.4 °C (Figure 4e,f), the trend is continued: The transition occurs at even lower pressures (30–40 MPa), the behavior is not fully reversible, and an additional shoulder at high *q*-values appears below. We note that, even though the trajectory at 35.4 °C appears to be close to the coexistence line (Figure 1), this is not reflected in the scattering data: The solution stays in the two-phase state during the entire run.

To summarize, at all investigated temperatures, a transition between small mesoglobules and large aggregates is seen, consistent with the observations from OM. The transition is the more pronounced, the closer the sample temperature is to the coexistence line. For all temperatures, the neutron scattering intensity—and hence the scattering contrast—decreases as the pressure is increased toward the transition pressure, i.e., the water content of the mesoglobules increases. Upon decreasing pressure, the water-rich large aggregates transform back into smaller, more polymer-rich mesoglobules, but the changes are not fully reversible. To quantify the sizes from the decay patterns and to exploit the overall intensity to characterize the behavior of the water content, we fitted the expressions in eqs 1 and 2, i.e., one Beaucage form factor or the sum of two Beaucage form factors to the scattering curves. In some cases, when the aggregates were larger than the resolution limit, the Porod term in eq 5 was used.

**Figure 5:**
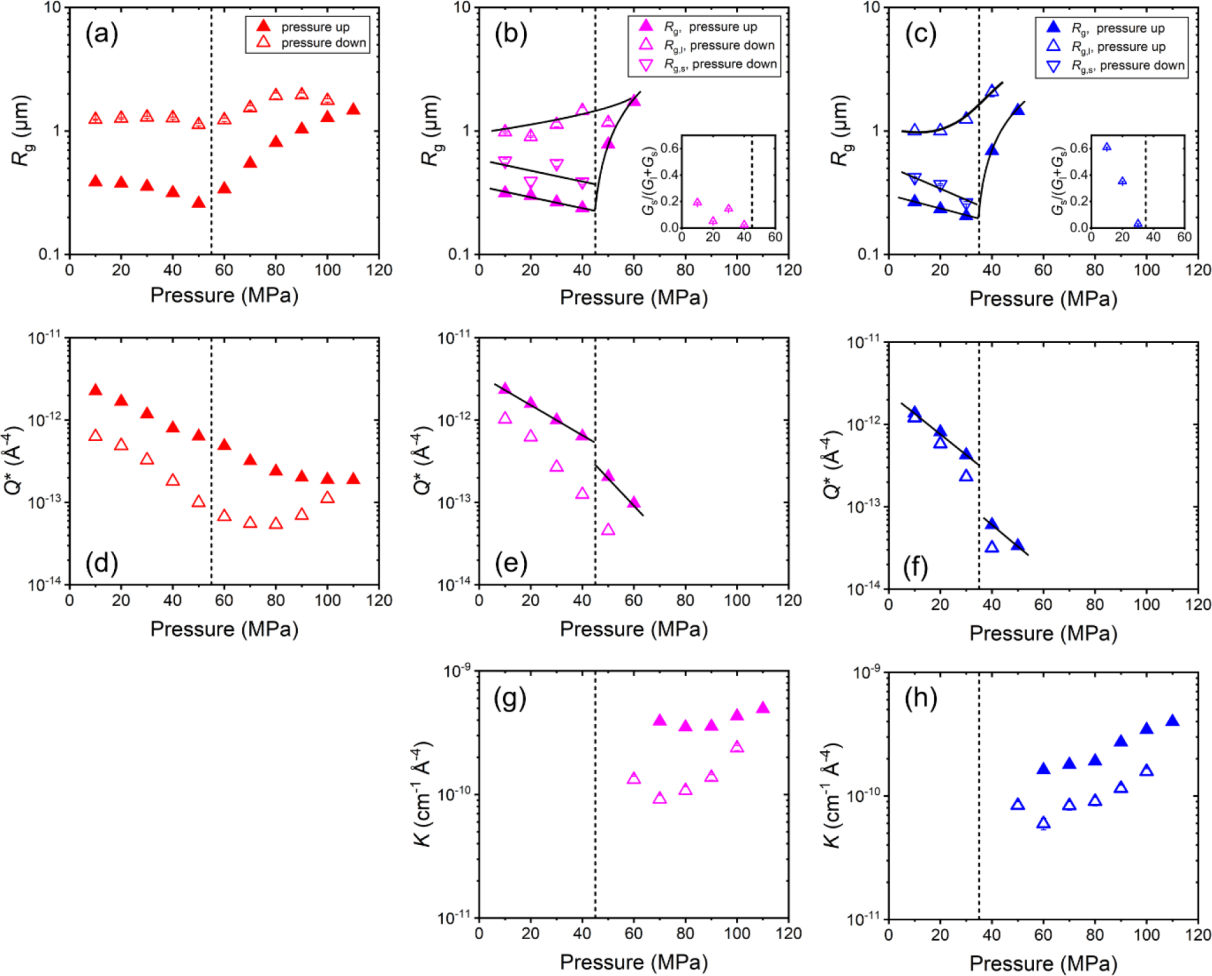
Pressure-dependent structural parameters obtained from model-fitting the VSANS data from Figure 4, i.e., during pressure scans at 37.0 °C (left), 36.2 °C (middle) and 35.4 °C (right) following protocol A. (a-c) Radii of gyration *R*_g_, *R*_g,l_ and *R*_g,s_, as indicated. In (b) and (c), the insets show the fraction of amplitudes *G*_s_/(*G*_s_+*G*_l_). (d-f) Invariant Q*, (g, h) Porod constant *K*. Closed symbols: increasing pressure, open symbols: decreasing pressure. The vertical dashed lines indicate the transition pressures identified from the behavior of *R*_g_ during the increase of pressure. The full lines guide the eye.

The data at 37.0 °C could be modeled with a single Beaucage form factor, both during the increase and the decrease of pressure. The fits are excellent throughout (Figure 4a,b). Only at the smallest *q*-values, slight deviations are observed during the pressure increase (Figure 4a), namely an upturn, especially at pressures at 50 MPa and above. This may be due to weak parasitic scattering, and these data points were not considered in the fits. The resulting radius of gyration of the mesoglobules, *R*_g_, decreases slightly from 0.39 μm at 10 MPa to 0.26 μm at 50 MPa, then it increases to 1.5 μm at 110 MPa (Figure 5a). Upon the decrease of pressure, *R*_g_ increases slightly, then decreases to 1.2 μm at 60 MPa and stays at this value down to 10 MPa. Hence, there is a distinct change of behavior at 55 MPa, both upon increase and decrease of pressure, and a growth of the mesoglobules with increasing pressure is observed, as expected. However, the behavior is only partially reversible. The slight initial increase of *R*_g_ upon the decrease of pressure indicates that the mesoglobules still grow, and that their shrinkage is delayed. The water content in the mesoglobules is accessible via the invariant Q*, which depends on the scattering contrast Δρ and the volume fraction of mesoglobules (eq 4). During the initial pressure increase, Q* decreases over the entire pressure range and levels off at 90 MPa (Figure 5d), which we assign mainly to an uptake of water, which reduces Δρ, while the volume fraction of aggregates presumably only changes marginally. Q* decreases further when pressure is decreased again and only starts to increase again at pressures below 80 MPa. The initial value at 10 MPa is not recovered. Thus, even during pressure decrease, the mesoglobules keep taking up water, until a pressure of 80 MPa is reached. Only below this pressure, they release water and shrink again. To conclude, at 37.0 °C, the mesoglobules take up water with increasing pressure and start to swell at 55 MPa. Since the size increase is rather modest, coalescence of the mesoglobules does not seem to play an important role. Upon the subsequent decrease of pressure, the mesoglobules retain a certain amount of water, which is possibly trapped at the interior of the mesoglobules, and hinders them in shrinking back to the original size. Apparently, the chain mobility is so low that, at this temperature, which is furthest away from the coexistence line, the structural changes require times that are longer than the measurement times.

At 36.2 °C, the data could be modeled with a single Beaucage form factor at pressures up to 60 MPa. The resulting structural parameters take similar values and behave similarly as at 37.0 °C (Figure 5b,e). However, at this temperature, *R*_g_ increases abruptly from 0.24 μm at 40 MPa to 0.78 μm at 50 MPa and continues to increase strongly, i.e., the transition pressure is located at 45 MPa. At 10 MPa, the value of the invariant Q* is similar to the one at 37.0 °C, but it decreases more strongly up to 40 MPa and decreases discontinuously at 45 MPa (Figure 5e). Thus, at the transition pressure of 45 MPa, the mesoglobules take up more water, and they grow abruptly, which may be assigned to the coalescence of the mesoglobules. The latter is more prominent than at 37.0 °C, because the chains are more hydrated and thus more mobile. Above 60 MPa, the Beaucage term could not be fitted any longer, because the shoulder in the scattering data vanishes, i.e., *R*_g_ is too large to be determined. Instead, the Porod term (eq 5) had to be used. The Porod constant *K* increases slightly between 70 and 110 MPa (Figure 5g). The Porod constant is indicative of the contrast, Δρ, and the specific surface *S*/*V* of the aggregates (eq 6), that, for spherical particles, is inversely proportional to the radius. Hence, an increase of *K* with pressure indicates an increase of contrast, i.e., water is repelled, and/or a decrease of the size. The expulsion of water from the mesoglobules seems more likely and may be due to the fact that the distance to the coexistence line is increased, as pressure is increased isothermally.

When pressure is decreased again at 36.2 °C, the large aggregates persist. *K* decreases with decreasing pressure, i.e., water is taken up and/or the aggregate size increases, and these changes are more pronounced than during the pressure increase. At 50 MPa and below, the Beaucage form factor could be used again for fitting the data, and *R*_g_ is found at 1.2 μm, which is slightly larger than the value obtained during the pressure increase. At 40 MPa and below, i.e., below the transition pressure, two Beaucage terms had to be used, giving *R*_g,l_ values of 0.9–1.5 μm and *R*_g,s_ values of 0.39–0.57 μm. *R*_g,s_ is similar, but a factor of 2 larger than the *R*_g_ values during the pressure increase. The invariant Q* follows the trend of the pressure increase, but does not recover the initial value. Thus, two types of structures are observed. The relative amplitude of the Beaucage term describing the scattering from the small structures, *G*_s_/(*G*_l_+*G*_s_), increases with decreasing pressure (inset of Figure 5b), but remains below 0.2. (While a strict interpretation of the value is not straightforward, because the amplitudes *G*_s_ and *G*_l_ depend on the amount, volume and scattering contrast of the structures, we still give the values for comparison, see below.) We conclude that, during the pressure increase at 36.2 °C, the mesoglobules take up water, and at 45 MPa, their water content is sufficiently high to enable coalescence, which results in large water-rich aggregates at higher pressures. On the way back, these large aggregates shrink, and smaller structures are formed again, but the large aggregates do not completely disappear. Thus, the transition back to small mesoglobules takes place, but a fraction of these mesoglobules is probably still connected and form large clusters. The shrinkage of these clusters may be due to a steady release of small mesoglobules and to an overall contraction and a release of water.

At 35.4 °C, similar behavior with increasing pressure is observed as at 36.2 °C (Figure 5c,f and h). The only difference is that the mesoglobules at the starting pressure of 10 MPa are smaller (*R*_g_ = 0.27 μm, Figure 5c), and the transition pressure is lower, namely 35 MPa. Moreover, the changes at the transition pressure are more pronounced, for instance, the decrease of the invariant at the transition pressure (Figure 5f). During the decrease of pressure, *G*_s_/(*G*_l_+*G*_s_) increases from ca. zero at 30 MPa to 0.6 at 10 MPa (inset of Figure 5c), i.e., the fraction of mesoglobules is larger than at 36.2 °C, which points to a stronger contraction of the chains and a higher degree of reversibility. Thus, during the pressure increase, the swelling, the water uptake and the coalescence occur more abruptly than at higher temperatures. During the pressure decrease, the smaller mesoglobules form to a higher extent than at higher temperatures, but they cannot completely separate from each other.

**Figure 6:**
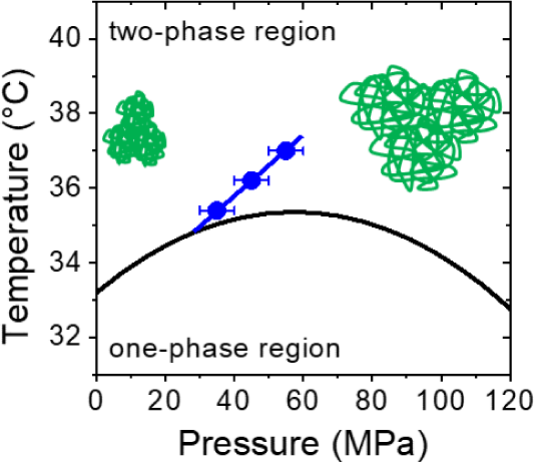
Temperature–pressure phase diagram of the investigated aqueous PNIPAM solution. Solid black line: coexistence line between the one- and two-phase state from Figure 1. Symbols: transition pressures determined from the scans during the increase of pressure. The tilted blue line is a guide to the eye. The cartoons depict the compact and small mesoglobules at low pressures and the swollen and large aggregates at high pressures.

**Figure 7:**
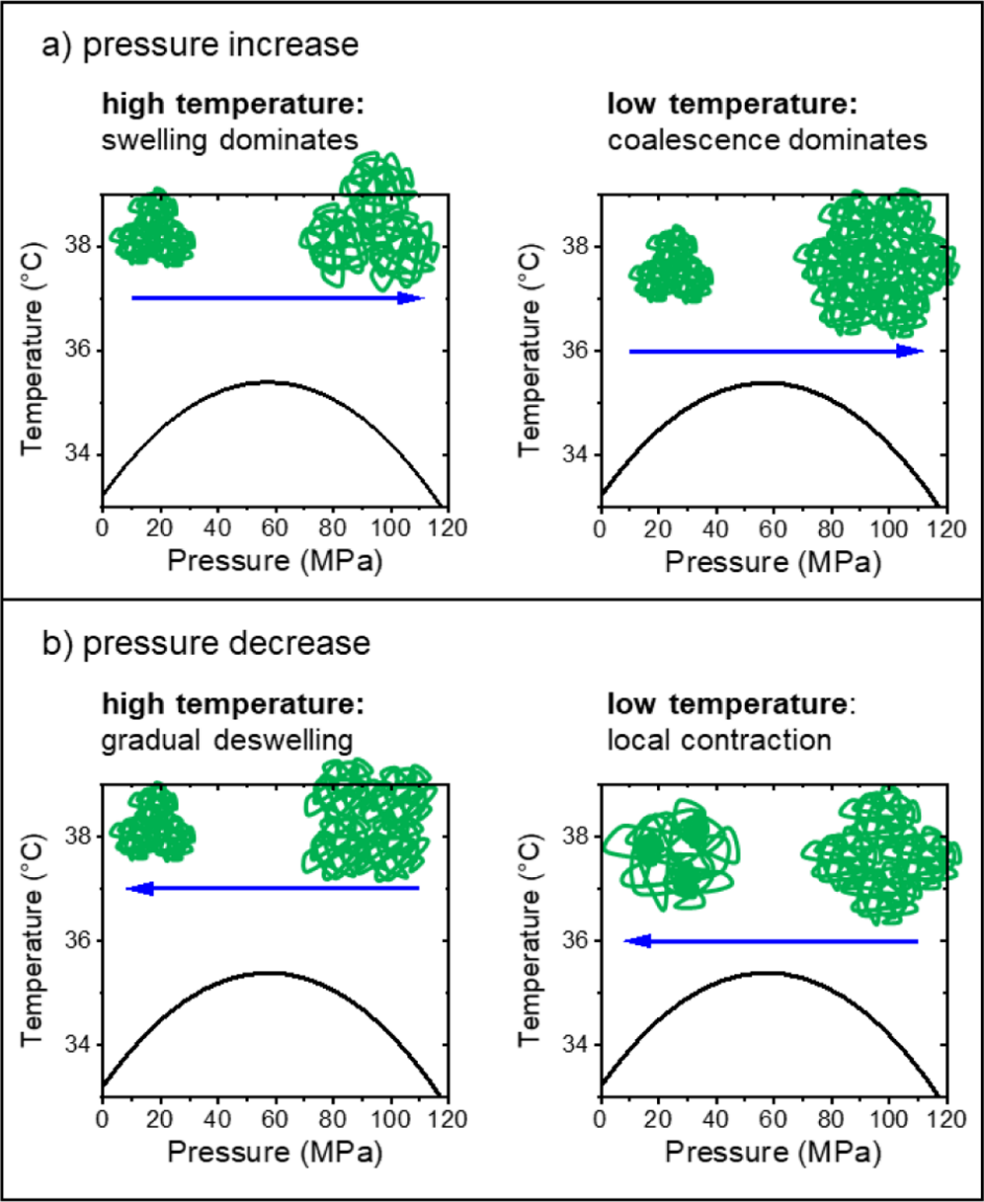
Schematic drawings of the processes identified during pressure increase (a) and decrease (b) following protocol A for high (37.0 °C) and low temperatures (36.2 and 35.4 °C).

The transition pressures between the compact mesoglobules at low pressures and swollen large aggregates at high pressures, as obtained from the three scans following protocol A, lie on a tilted line (Figure 6). The line connecting the transition pressures meets the coexistence line—which separates the one-phase and the two-phase state—on the left side of the maximum of the coexistence line, i.e., the newly identified transition line and the maximum do not appear to be directly related. While the transition between mesoglobules and aggregates is continuous at the highest temperature measured and mainly due to swelling, it becomes more and more abrupt as temperature is decreased, and here, coalescence dominates (Figure 7a). We attribute this difference to the higher degree of hydration and the resulting enhanced chain mobility at the lower temperatures, that are closer to the coexistence line, which makes coalescence possible. In computer simulations of single chains, a transition between collapsed, dehydrated chains at low pressures and a hydrated globular state was found in the two-phase state as well, however, the transition pressure was independent of temperature, i.e., the transition line was vertical.^29^

The transition is, however, not completely reversible, which is probably due to kinetic effects (Figure 7b). At the highest temperature, small and compact mesoglobules form again upon decreasing the pressure, presumably by deswelling and contraction of the chains, and these mesoglobules are larger than the initial ones. At the two lower temperatures, the scattering from the large aggregates does not fully vanish upon decreasing the pressure, and we conclude that the compact mesoglobules cannot fully separate from each other and stay partially connected. Thus, while the enhanced chain mobility at these lower temperatures allows the formation of mesoglobules, the complete disintegration of the large aggregates is slower than the time scale of the experiment.

#### An Experiment for Exploring Nonequilibrium States

The deswelling and disintegration process of the large aggregates during the pressure decrease may be affected by mesoglobules or compact nanodomains that have not fully dissolved during the initial increase of the pressure. To eliminate such structures, we carried out a pressure scan following protocol B, i.e., after the initial pressure increase at 35.4 °C to 100 MPa (identical to protocol A), the sample was cooled to 28.8 °C at this pressure, was kept there for 1 h and was subsequently reheated to 35.4 °C, before decreasing the pressure again.

**Figure 8:**
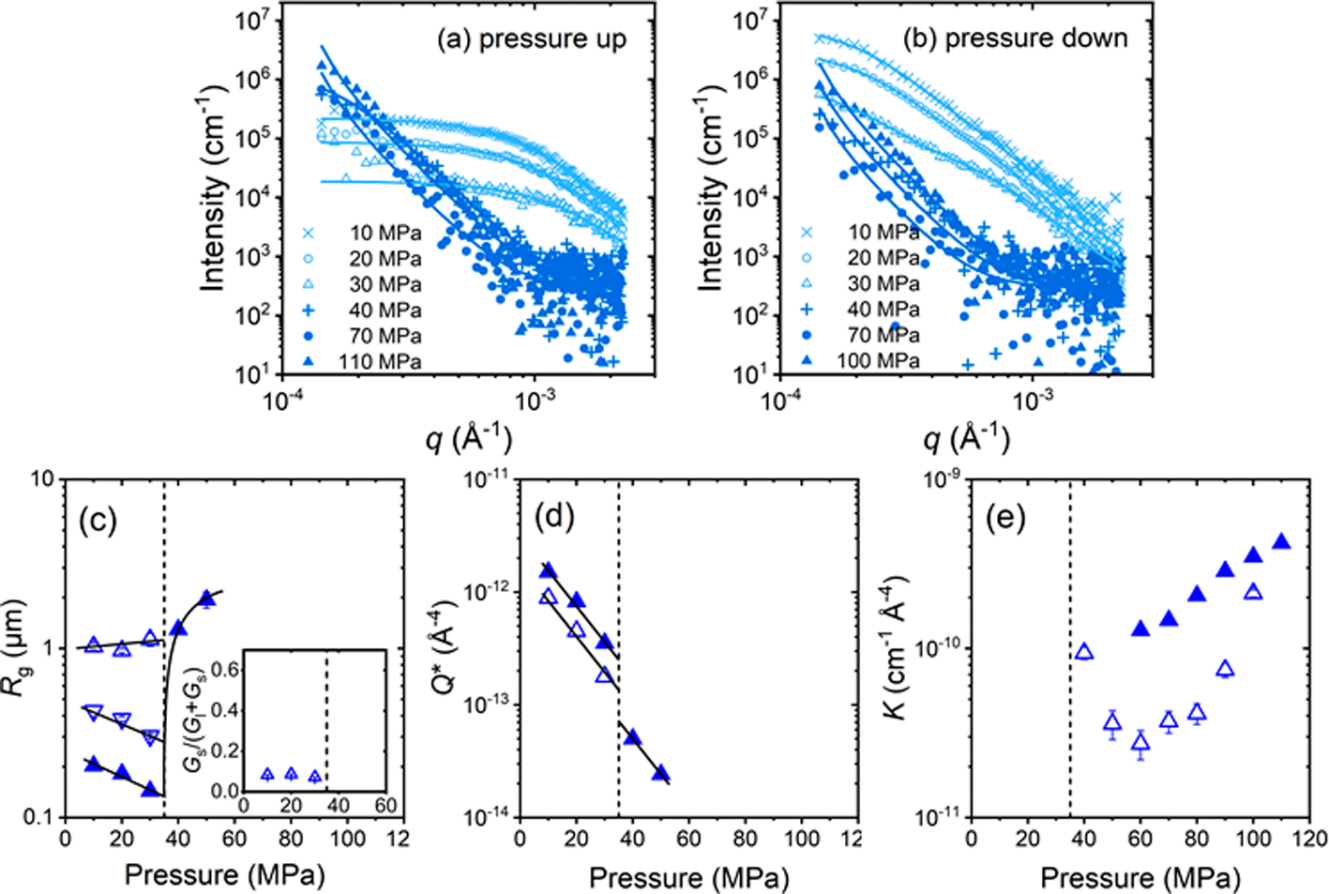
Representative VSANS curves obtained during protocol B during a pressure increase from 10 to 110 MPa (a), and from 100 to 10 MPa (b) in steps of 10 MPa at 35.4 °C, as described in the text. Symbols: experimental data. Solid lines: fits of 1 or 2 Beaucage form factors or of the Porod form factor (eqs 1, 2 and 5). (c-e) Resulting pressure-dependent structural parameters: (c) radius of gyration *R*_g_, (d) the invariant Q*, and (e) the Porod constant *K*. Same notation as in Figure 5.

Figure 8a,b show the resulting scattering curves for increasing and decreasing pressure. During pressure increase, the curves look very similar to the ones obtained following protocol A, and the transition is found at the same pressure of 35 MPa, which demonstrates the reproducibility of the structures. The same holds for the decrease of pressure down to 30 MPa. However, below 30 MPa, the intensity of the shoulder at low *q*-values is higher than in protocol A, i.e., the fraction of large aggregates is larger.

The data were analyzed in the same way as the ones from protocol A at 35.4 °C. While the values of *R*_g,s_ during the increase of pressure differ slightly from the ones in protocol A, they are equal during the pressure decrease, and the transition pressure is the same, namely 35 MPa. *G*_sd_/(*G*_mg_+*G*_sd_) is 0.08–0.1 during the pressure decrease from 30 to 10 MPa. These values are significantly smaller than in protocol A, i.e., the small mesoglobules do not form to the same extent. Possibly, in protocol A, some of them were a remainder of small, compact regions that did not hydrate well during the time spent at high pressure and served as nucleation centers for the reformation of the small mesoglobules during pressure decrease. The invariant Q* is the same throughout. The Porod constant *K* is the same for increasing pressure, but is lower for protocol B for the decrease of pressure from 110 to 50 MPa.

To conclude, far less mesoglobules emerge during pressure decrease from homogeneous large and water-swollen aggregates, which are formed by heating from the one-phase state (protocol B). This is different when starting from possibly still slightly inhomogeneous large aggregates (protocol A). Still, the overall behavior and the location of the transition are unaltered.

## Conclusions

In the present study, we investigate dispersions of mesoglobules formed by a semidilute aqueous solution of PNIPAM in the two-phase state above the cloud point temperature. We focus on the transition between the small mesoglobules at low pressures and the large aggregates at high pressures. High-pressure optical microscopy reveals an increase of the mesoglobule size with increasing pressure. The size and water content are characterized using high-pressure very small angle neutron scattering at temperatures above the (elliptical) coexistence line in the temperature–pressure frame. The sharpness of the transition between these regimes depends on temperature, i.e., on the distance to the coexistence line and thus on the degree of hydration and the chain mobility. At high temperatures, i.e., far away from the coexistence line, the transition is continuous and mainly proceeds via swelling of the mesoglobules, as pressure is increased. At low temperatures, i.e., close to the coexistence line, the transition is abrupt and involves coalescence of swollen mesoglobules as well. The transition pressure increases with temperature. Upon decreasing the pressure, small compact domains form, which have the same size as the initial mesoglobules, however, they cannot fully separate from each other. To test whether, during the increase of pressure across the transition line, small compact domains persist, that serve as nucleation centers for chain contraction during the subsequent pressure increase, we carried out a scan at a low temperature and brought the sample into the one-phase state before decreasing the pressure again. This results in a complete disintegration of the large aggregates into a semidilute PNIPAM solution. During the subsequent reformation of large aggregates upon heating and the stepwise pressure decrease, the same behavior was found as without the dissolution, but the number of small compact domains is significantly smaller than in the purely isothermal scans.

Our findings are in qualitative agreement with atomistic simulations on a single PNIPAM chain that revealed a transition between collapsed, dehydrated chains at low pressures and a hydrated globular state at higher pressures.^29^ This change of hydration was tentatively assigned to the decrease of the intrachain hydrophobic contacts with increasing pressure, as evident from the decrease of the position of the peak in the radial distribution function between C atoms of side chain methyl groups and water oxygen.^29^ However, for the single chain, a transition pressure of ca. 200 MPa was found, independent of pressure, which is significantly higher than in the semidilute solution. Though the authors of ref (29) intended the phase diagram as a schematic illustration due to the limited number of state points, it is remarkably similar to the data from experiments. A possible reason for the discrepancies could be that the mesoglobules consist of a large number of chains, and not only the hydration behavior of the single chain, but also their disentanglement and diffusion come into play. Especially at low temperatures, not only swelling of the existing mesoglobules is observed, but also their coalescence, which seems to be enabled by the enhanced chain mobility, which increases with decreasing temperature, since it mainly depends on the degree of hydration of the chains, i.e., the proximity to the coexistence line.

The pressure dependence of the degree of hydration and the resulting differences in chain mobility in the PNIPAM system in the two-phase state offers the possibility to investigate the transition processes between compact mesoglobules and water-rich large aggregates and back. Depending on the path in the *p*-*T*-plane, coalescence is favored or not, which allows tuning the particle size. On the way back, entanglements seem to hamper the separation of small compact domains from each other. These results provide a fundamental new insight into the phase behavior of aqueous PNIPAM solutions at variable pressure, which may be extrapolated to more complex polymeric systems.
